# General population reference values for the Functional Assessment of Cancer Therapy‐Lung and PROMIS‐29

**DOI:** 10.1002/cam4.5920

**Published:** 2023-05-06

**Authors:** Nisha A. Mohindra, John Devin Peipert, Steven I. Blum, James W. Shaw, John R. Penrod, David Cella

**Affiliations:** ^1^ Division of Hematology and Oncology, Department of Medicine Northwestern University Feinberg School of Medicine Chicago Illinois USA; ^2^ Robert H. Lurie Comprehensive Cancer Center Northwestern University Chicago Illinois USA; ^3^ Department of Medical Social Sciences Northwestern University Feinberg School of Medicine Chicago Illinois USA; ^4^ Bristol Myers Squibb Princeton New Jersey USA

**Keywords:** lung cancer, patient‐reported outcomes

## Abstract

**Background:**

Therapeutic advances in lung cancer have turned attention toward patient‐reported outcome measures (PROMs) as important clinical outcomes. The Functional Assessment of Cancer Therapy‐Lung (FACT‐L) is a common endpoint in lung cancer trials. This study calculated FACT‐L reference values for the United States (US) general population.

**Methods:**

Adults from the US general population (*N* = 2001) were surveyed between September 2020 and November 2020. Surveys contained 126 questions, including the FACT‐L [36 items; FACT‐G and four subscales (Physical Well‐Being [PWB], Social Well‐Being [SWB], Emotional Well‐Being [EWB], and Functional Well‐Being [FWB]) and the Lung Cancer Subscale (LCS), and a Trial Outcome Index (TOI)]. Reference values for each FACT‐L scale were calculated with means for the total sample and separately for participants with: no comorbidities, COVID‐19 as only comorbidity, no COVID‐19.

**Results:**

In the total sample, the reference scores were as follows: PWB = 23.1; SWB = 16.8; EWB = 18.5; FWB = 17.6; FACT‐G = 76.0; LCS = 23.0, TOI = 63.7, and FACT‐L Total = 99.0. Scores were lower for those reporting a prior diagnosis of COVID‐19, especially for SWB (15.7) and FWB (15.3). SWB scores were lower than previous references values.

**Conclusions:**

These data provide US general adult population reference value set for FACT‐L. While some of the subscale results were lower than those found in the reference data for other PROMs, these data were obtained in a more contemporaneous time frame juxtaposed with the COVID‐19 pandemic and may represent a new peri‐pandemic norm. Thus, these reference values will be useful for future clinical research.

## Abstract 2

This study provides reference values for FACT‐L and its subscales from the general population, with most scores similar to those from previous references value sets. These reference values will be useful for future clinical research and will help us understand the extent of burden related to treatment relative to the population at large.

## 1 INTRODUCTION

Lung cancer is the second most commonly diagnosed malignancy among men and women in the United States (US) and remains the leading cause of cancer related deaths in the United States and worldwide.^1^, ^2^ Therapeutic advancements in lung cancer have led to meaningful improvements in survival for patients, namely in the form of novel, molecularly targeted agents and immunotherapy agents.^3^ With these advances, we are now seeing declines and delays in mortality rates associated with lung cancer,^3^ yet we seek to further understand the impact of these agents on health‐related quality of life (HRQoL) amidst this changing treatment paradigm.

The US Food and Drug Administration (FDA) issued a final guidance in 2009 for the use of patient‐reported outcome (PRO) instruments to support claims in approved medical product labeling,^4^ and there has been a parallel increase in measuring PROs in clinical trials.^5^ There has also been discussion of how to implement PRO measures for symptom monitoring or for reimbursement as an aspect of value‐based care.^5^, ^6^

The Functional Assessment of Cancer Therapy (FACT)^7^ measurement system is a family of cancer‐targeted HRQOL and symptom measures. FACT measures are available for a variety of tumor sites (e.g., breast, lung, colon, prostate, and kidney), and these measures cover multiple HRQOL domains (e.g., symptoms, physical well‐being, and emotional well‐being). The FACT‐Lung (FACT‐L) measure^8^ is a lung cancer‐targeted PRO measure developed using a rigorous, multi‐stage process wherein items were generated and evaluated by a group of lung cancer patients and lung cancer care providers.

Several prior reports have demonstrated the reliability and validity of the FACT‐L, including its subscales.^8^, ^9^, ^10^ Despite its well‐documented psychometric properties, there remains a need for additional guidance on the clinical interpretation of FACT‐L responses and scores. Population reference values, gathered on a sample of the US adult general population, can guide clinicians and other interested investigators in FACT‐L score interpretation. General population reference values are important because one might otherwise assume a reference score should be a perfect score which is not the case. Understanding general population values allows for a more realistic assessment of “ceiling” values. Therefore, the objective of this study was to provide US population reference values for the FACT‐L questionnaire and its subscales.

## 2 METHODS

### 2.1 Participants and survey procedure

We recruited 2001 participants from the Focus Pointe Global (FPG, now Schlesinger Quantitative) Internet panel of the US general population using a quota sample procedure. The sample was drawn from an opt‐in panel of 1.6 million members. Approximately 21% of panel members are from the Western US, 32% from the Midwest, 20% from the South, and 27% from the Northeast; approximately 60% are female and 40% are male. Individuals were eligible for participation in this study if they were as follows: (1) able to understand and willing to sign written informed consent in English and (2) aged ≥18 years old at time of enrollment. Potentially eligible participants for our study completed a screener profile, then were called by the Schlesinger recruiting team to confirm their answers on the phone match the answers on the screener and to assess their ability to complete surveys. Once they passed the phone screening, they were sent a confirmation letter and invited to participate in the study. They were then called by the Schlesigner verification team and spoken with again to confirm eligibility.

Our study sample was designed to match the joint distribution of age and gender in the US general adult population. The population was divided into two gender groups (male and female) and 15 age categories: 18–19, 20–24, 25–29, 30–34, 35–39, 40–44, 45–49, 50–54, 55–59, 60–64, 65–69, 70–74, 75–79, 80–84, and ≥85 years of age. The proportion of each gender within each of these categories within the US adult general population as represented in the 2017 American Community Survey (ACS) 1‐year estimates was determined (see Table S1). Then, quotas were created to represent these proportions of 2000 participants. Eligible individuals were asked to complete a survey consisting of 126 questions (which consisted of the FACT‐L and other PRO measures that were co‐administered) following completion of online consent. Participants were randomized to receive one of two orders of measure administration within the survey (see Appendix S1). Individuals meeting criteria for each quota were sought until the quota was filled. Participants were recruited and completed surveys between September and November 2020. The time frame of the study occurred during the COVID‐19 pandemic, and there was a subset of participants who answered “yes” to ever being told by a health professional that they had COVID‐19 or the novel Corona virus. The timeframe between the COVID‐19 infection and filling out these surveys for those participants is not known. Interpretation of these values have been included in an exploratory fashion. After collecting the survey data, a de‐identified dataset was shared with investigators at Northwestern University for analysis. A human‐subjects protocol was submitted to the Northwestern University Institutional Review Board (IRB) for this study and was determined to be exempt from review given the de‐identification of data (STU00209906).

### 2.2 Measures

The primary measure of interest for this study was the FACT‐L, including all of its subscales. The FACT‐L is a 36‐item questionnaire that adds a 9‐item Lung Cancer Subscale (LCS; 7 items of which are scores as LCS) to the 27‐item FACT‐G, a general cancer HRQoL questionnaire with subscales to measure Physical Well‐Being (PWB; 7 items), Social/Family Well‐Being (SWB; 7 items), Emotional Well‐Being (EWB; 6 items), and Functional Well‐Being (FWB; 7 items) subscales. In addition to the FACT‐G total score and its subscale components, the LCS, PWB, and FWB are combined to create the FACT‐L Trial Outcome Index (TOI; PWB + FWB + LCS subscale; 21 items) and the FACT‐G and LCS are combined to create a FACT‐L Total score (FACT‐G + LCS subscale; 36 items). Each item in the instrument has five response options: “Not at all” (0), “A little bit” (1), “Somewhat” (2), “Quite a bit” (3), and “Very much” (4). The standard FACT‐L scoring method was used to create scores for each scale and subscale, which entails creating a prorated sum of item responses, resulting in the following possible ranges: 0–28 for the PWB, SWB and FWB subscales; 0–24 for EWB; 0–108 for the FACT‐G total score; 0–28 for the LCS; 0–84 for the FACT‐L TOI; and 0–136 for the FACT‐L Total score. For all scales, higher scores indicate better HRQoL. To achieve this interpretation, before item responses were combined, negatively worded items were reverse‐coded. In addition, missing FACT‐L item responses were accounted for in the scoring of scales by prorating when >50% of the items in a scale were not missing for subscales and >80% were not missing for composite scales, including the FACT‐G, FACT‐L Total, and the FACT‐L TOI.

In addition to the FACT‐L, our survey included several additional questionnaires to help characterize participants' health. These included the Patient‐Reported Outcomes Measurement Information System® (PROMIS®) 29+2 Item Health Profile v2 (PROMIS‐29+2). The PROMIS‐29+2 assesses seven domains of HRQoL common to all PROMIS profiles with four‐item short forms (physical function, anxiety, depression, fatigue, sleep disturbance, ability to participate in social roles and activities, and pain interference),^11^ a single numeric rating scale item for pain intensity, and two additional items on cognitive function. PROMIS domain scores are on a *T* score metric with a mean of 50 and standard deviation of 10, referenced to the US general population. The addition of the cognitive function items to the standard PROMIS‐29 profile allows for the calculation of the PROMIS‐Preference (PROPr) score,^12^ which ranges from −0.022 to 1 and uses preferences generated from a nationally representative US sample. We assessed the self‐reported Eastern Cooperative Oncology Group performance status rating (ECOG PSR), which categorized participants as (0) normal activity without symptoms, (1) some symptoms but do not require bed rest during the waking day, (2) require bed rest for <50% of the waking day, (3) require bed rest for more than 50% of the waking day, and (4) unable to get out of bed.^13^ We also included three patient global impression of severity (PGIS) items to assess common lung cancer symptoms: “Please choose the response below that best describes the severity of your fatigue over the past week,” “Please choose the response below that best describes the severity of your pain over the past week,” “Please choose the response below that best describes the severity of your shortness of breath over the past week.” For each of the PGIS items, the response options were as follows: “None,” “Mild,” “Moderate,” “Severe,” and “Very Severe.” The survey asked participants whether they had been told by a doctor that they had any of 23 comorbid conditions, including COVID‐19. Finally, we asked about several demographic questions to characterize the recruited sample of participants, including their race, ethnicity, current marital status, highest level of education completed, and employment status.

### 2.3 Statistical analysis

Participants' characteristics, including each comorbid condition and the total number of comorbid conditions, were summarized with frequencies and proportions or means, standard deviations (SD), and ranges, as appropriate. Where possible, we summarized these characteristics for the US general adult population using the 2017 ACS 1‐year estimates as well as the survey data. Reference values for each FACT‐L scale were calculated with means, SDs, minimum observed scores, maximum observed scores, and scores at the 5th, 25th, 50th (median), 75th, and 95th percentiles. In addition, we calculated the percent and frequency of each score at its possible minimum (floor) and maximum (ceiling) value. We also calculated the internal consistency reliability of each scale with Cronbach's alpha coefficients, which were interpreted using the following standards: ≥0.70 = acceptable, ≥0.80 = good, and ≥0.90 = excellent.^14^ We calculated 95% confidence intervals for Cronbach's alphas using psych package in R.^15^ Each of these reference value calculations were conducted for the total participant sample, for those reporting no comorbidities, for those reporting no COVID‐19 diagnosis, and those reporting a COVID‐19 diagnosis. To further characterize the sample's HRQOL, we calculated the mean and SD for each PROMIS‐29 domain, as well as the PROPr score. Finally, we tested the known‐groups validity of the FACT‐G, LCS, TOI, and FACT‐L Total scores. This procedure involved calculating and comparing mean scale scores between the groups of several anchor variables, including ECOG PSR (0 vs. 1 vs. 2–4),^16^ number of comorbidities (0 vs. 1 vs. 2 vs. 3 vs. ≥4), and the PGIS items for shortness of breath, fatigue, and pain. We used one‐way analysis of variance (ANOVA) models with least squares (LS) means to determine whether the mean LCS, TOI, and FACT‐L Total scores were significantly different between adjacent categories of each of the anchors. A *p*‐value of <0.05 was considered statistically significant. We also calculated standardized effect sizes for these comparisons as the adjacent group mean difference divided by the pooled scale SD. The magnitude of effects was interpreted according to the following standards: ≥0.20 to <0.50 = small; ≥0.50 to <0.80 = medium; and ≥0.80 = large.^17^

## 3 RESULTS

In total, 4888 individuals were invited to participate based on initial assessment of eligibility and opened the survey. Of these, 171 were subsequently determined to be ineligible based on responses to screening questions, 1775 begun but did not complete the survey, and 941 completed a survey after their particular stratum for recruitment had already closed. This left 2001 participants for analysis. The joint distribution of the sample's age and gender matched that of the US general population per the quotas set for the study (Table S1). Additional participant characteristics are shown in Table 1. The largest proportion of participants were White (*n* = 1500, 75%), married (*n* = 862, 43.1%), had a University or Post‐Graduate Degree (*n* = 801, 40.0%), and were employed full‐time (*n* = 922, 46.1%). A large majority had an ECOG PSR of 0 (normal activity without symptoms; *n* = 1305, 65.2%). In general, the distribution of these additional demographic characteristics also matched the 2017 ACS 1 Year estimates for the US adult population. Two exceptions were that the proportions of Latino/Hispanic participants and married participants in our survey were lower than the US general population. We note that the survey was administrated in English only, likely accounting for the lower proportion of Latino/Hispanic participants. Prevalence of comorbid conditions is described in Table 2. Notably, 81 participants (4.1%) reported a COVID‐19 diagnosis. During the timeframe of this study, the rate of positive COVID‐19 diagnostic tests ranged from 4.8% to 11.9%, making the sample representative of the US population at that timeframe.^18^ It is also possible that COVID‐19 was under‐reported in our study due to stigma or lack of knowledge, which were pervasive at the time of the survey. Other common comorbidities were high blood pressure (*n* = 712, 35.6%), anxiety (*n* = 688; 34.4%), depression (*n* = 631, 31.5%), and arthritis or rheumatism (*n* = 463, 23.1%). Chronic lung disease (COPD), chronic bronchitis, or emphysema was reported by 136 (6.8%) of the participants. Multi‐morbidity was common, as 581 (29.0%) reported four or more comorbidities.

**TABLE 1:** Participant characteristics.

	Survey sample (*N* = 2001)	2017 American Community Survey adult population (*N* = 252,155,280)
Gender, *n* (%)		
Female	1031 (51.5%)	131,074,644 (51.3%)
Male	970 (48.5%)	124,252,949 (48.7%)
Age, median (range)	47 (18–92)	46 (18–96)
Race, *n* (%)		
White alone	1500 (75.0%)	186,530,101 (74.0%)
Black/African American alone	252 (12.5%)	31,101,918 (12.3%)
American Indian/Alaska native alone	10 (0.5%)	1,648,034 (0.7%)
Asian alone	123 (6.2%)	14,640,241 (5.8%)
Native Hawaiian/Pacific Islander alone	0 (0%)	436,188 (0.2%)
Other race alone	0 (0%)	11,611,710 (4.6%)
Two or more races	56 (2.8%)	5,866,015 (2.3%)
Missing	60 (3.0%)	‐
Latino/Hispanic ethnicity, *n* (%)	182 (9.1%)	40,310,228 (16.0%)
Marital status, *n* (%)		
Married	862 (43.1%)	126,532,032 (50.2%)
Never married	571 (28.5%)	76,508,327 (30.3%)
In a committed relationship	240 (12.0%)	‐^a^
Divorced	207 (10.3%)	28,892,416 (11.5%)
Widowed	90 (4.5%)	15,140,475 (6.0%)
Separated	31 (1.6%)	5,082,030 (2.0%)
Education level, *n* (%)		
Less than high school/secondary school	8 (0.4%)	30,425,348 (12%)
High school/secondary school	464 (23.2%)	65,015,745 (26%)
College or vocational school	728 (36.4%)	124,573,686 (49%)
University or post‐graduate degree	801 (40.0%)	27,464,753 (11%)
Employment, *n* (%)		
Employed (any employment)	1174 (59%)	154,385,876 (61%)
Unemployed	146 (7%)	8,243,807 (3%)
Not in labor force	681 (34%)	89,525,597 (36%)
ECOG performance status rating		
0—Normal activity without symptoms	1305 (65.2%)	‐
1—Some symptoms but do not require bed rest during the waking day	548 (27.4%)	‐
2—Require bed rest for less than 50% of the waking day	118 (5.9%)	‐
3—Require bed rest for more than 50% of the waking day	27 (1.4%)	‐
4—Unable to get out of bed	3 (0.2%)	‐

^a^ This category was not given in ACS 2017.

**TABLE 2:** Prevalence of comorbid conditions (*N* = 2001).

Total number of comorbid conditions, *n* (%)	
0	379 (18.9%)
1	374 (18.7%)
2	353 (17.6%)
3	314 (15.7%)
4+	581 (29.0%)
Prevalence of specific comorbid conditions	
COVID‐19	81 (4.1%)
High blood pressure (hypertension)	712 (35.6%)
Angina or chest pain	149 (7.5%)
Coronary artery disease	92 (4.6%)
Heart failure or congestive heart failure	69 (3.5%)
Heart attack or myocardial infarction	78 (3.9%)
Stroke, brain hemorrhage, or transient ischemic attack	70 (3.5%)
Liver disease, hepatitis, or cirrhosis	65 (3.3%)
Kidney (renal) disease	79 (4.0%)
Arthritis or rheumatism	463 (23.1%)
Diabetes or high blood sugar or sugar in urine	333 (16.6%)
Osteoarthritis or rheumatoid arthritis	337 (16.8%)
Cancer (other than non‐melanoma skin cancer)	164 (8.2%)
Asthma	349 (17.4%)
Chronic lung disease (COPD), or chronic bronchitis, or emphysema	136 (6.8%)
Migraines or severe headaches	433 (21.6%)
Depression	631 (31.5%)
Anxiety	688 (34.4%)
Alcohol or drug problem	92 (4.6%)
HIV or AIDS	41 (2.1%)
Spinal cord injury	51 (2.6%)
Multiple sclerosis	41 (2.1%)
Sleep disorder	382 (19.1%)

Table 3 shows the reference values for FACT‐L scales (LCS, TOI, and FACT‐L Total) in the total sample, those with no comorbidities, those without a COVID‐19 diagnosis, and those with COVID‐19 diagnosis. For the lung cancer‐targeted scales, in the total sample, the mean scores were 23.0 (LCS), 63.7 (TOI), and 99.0 (FACT‐L Total). Mean scores were comparatively higher in the sample with no comorbidities (LCS = 25.1, TOI = 72.0, FACT‐L Total = 110.1). Compared to participants without a COVID‐19 diagnosis, those with a COVID‐19 diagnosis had consistently lower scores for all FACT‐L scales and the FACT‐G Total score. This trend was reflected in the PROMIS‐29 and PROPr scores as well (Table 4). While PROMIS‐29 and PROPr scores for participants without COVID‐19 were similar to those from the general population, mean scores for participants with COVID‐19 tended to be 2–3 points worse, on average. These values are generally comparable to previous reference values published by Brucker and colleagues from the US general population for the FACT‐G, with the exception of the SWB scale.^19^ The reference values from the current study are visualized in comparison with those from the Brucker study in Table 5.

**TABLE 3:** Reference values for FACT‐L scales in US adult population.

	PWB (0–28)	SWB (0–28)	EWB (0–24)	FWB (0–28)	FACT‐G (0–108)	LCS (0–28)	FACT‐L Total (0–136)	TOI (0–84)
*Total sample (N = 2001)*								
Mean	23.1	16.8	18.5	17.6	76.0	23.0	99.0	63.7
Standard deviation	5.1	7.1	5.1	6.7	19.8	4.4	23.0	14.1
Percent at floor	0.1%	0.9%	0.1%	0.4%	0%	0%	0%	0%
Percent at ceiling	14%	6.1%	14.0%	5.4%	1%	12.6%	0.7%	2.2%
Minimum observed score	0.0	0.0	0.0	0.0	9.0	3.0	20.0	11.0
5th percentile	12.0	4.0	8.0	5.0	40.0	14.0	57.0	37.0
25th percentile	21.0	11.7	16.0	13.0	63.0	21.0	84.0	55.0
Median	25.0	17.5	20.0	18.0	79.0	24.0	103.0	66.0
75th percentile	27.0	22.0	22.0	23.0	92.0	26.0	117.0	75.0
95th percentile	28.0	28.0	24.0	28.0	103.8	28.0	130.0	82.0
Maximum observed score	28.0	28.0	24.0	28.0	108.0	28.0	136.0	84.0
Cronbach's alpha (95% confidence intervals)	0.88 (0.88, 0.89)	0.85 (0.84, 0.86)	0.85 (0.84, 0.86)	0.87 (0.86, 0.88)	0.94 (0.94, 0.94)	0.77 (0.76, 0.77)	0.94 (0.94, 0.94)	0.92 (0.91, 0.92)
*No comorbidities (N = 364)*								
Mean	26.2	17.1	21.1	20.7	85.0	25.1	110.1	72.0
Standard deviation	2.7	7.0	3.0	5.9	14.8	3.4	17.2	10.1
Percent at floor	0.0%	1.7%	0.0%	1.1%	0.0%	0.0%	0.0%	0.0%
Percent at ceiling	36.0%	9.1%	27.8%	13.7%	3.0%	27.5%	2.2%	7.4%
Minimum observed score	5.0	0.0	7.0	0.0	18.0	4.0	22.0	12.0
5th percentile	22.0	5.0	15.0	10.0	57.0	18.0	78.7	53.0
25th percentile	26.0	12.0	20.0	17.0	76.0	24.0	101.0	67.0
Median	27.0	18.0	22.0	21.5	86.0	26.0	112.0	74.0
75th percentile	28.0	22.0	24.0	25.0	96.7	28.0	124.0	79.0
95th percentile	28.0	28.0	24.0	28.0	106.0	28.0	133.0	84.0
Maximum observed score	28.0	28.0	24.0	28.0	108.0	28.0	136.0	84.0
Cronbach's alpha (95% confidence intervals)	0.85 (0.83, 0.87)	0.83 (0.80, 0.85)	0.75 (0.71, 0.79)	0.85 (0.83, 0.87)	0.91 (0.90, 0.92)	0.78 (0.75, 0.81)	0.93 (0.92, 0.94)	0.90 (0.88, 0.91)
*No COVID‐19 diagnosis (N = 1920)*								
Mean	23.2	16.8	18.6	17.7	76.3	23.1	99.4	64.0
Standard deviation	5.0	7.1	5.1	6.7	19.7	4.3	22.8	13.9
Percent at floor	0.1%	0.9%	0.1%	0.4%	0.0%	0.0%	0.0%	0.0%
Percent at ceiling	13.8%	6.3%	14.3%	5.5%	1.0%	13.0%	0.7%	2.2%
Minimum observed score	0.0	0.0	0.0	0.0	9.0	3.0	20.0	11.0
5th percentile	12.5	4.0	8.0	5.0	41.0	14.0	57.0	38.0
25th percentile	21.0	11.7	16.0	13.0	63.0	21.0	84.0	55.0
Median	25.0	18.0	20.0	18.5	79.0	24.0	103.0	67.0
75th percentile	27.0	22.2	23.0	23.0	92.0	26.0	117.5	75.0
95th percentile	28.0	28.0	24.0	28.0	104.0	28.0	130.0	82.0
Maximum observed score	28.0	28.0	24.0	28.0	108.0	28.0	136.0	84.0
Cronbach's alpha (95% confidence intervals)	0.88 (0.87, 0.89)	0.85 (0.84, 0.86)	0.85 (0.84, 0.86)	0.87 (0.86, 0.88)	0.94 (0.94, 0.94)	0.76 (0.74, 0.77)	0.94 (0.94, 0.94)	0.92 (0.91, 0.92)
*COVID‐19 diagnosis (N = 81)*								
Mean	21.2	15.7	17.1	15.3	69.4	19.3	88.7	55.8
Standard deviation	6.4	6.7	5.5	6.9	20.3	5.6	24.6	16.3
Percent at floor	0.0%	0.0%	0.0%	0.0%	0.0%	0.0%	0.0%	0.0%
Percent at ceiling	9.9%	2.5%	7.4%	3.7%	0.0%	3.7%	0.0%	0.0%
Minimum observed score	4.0	3.0	4.0	1.0	26.0	6.0	36.0	19.0
5th percentile	7.0	6.0	7.0	5.0	37.0	11.0	52.0	27.0
25th percentile	18.0	10.0	14.0	10.0	52.0	14.0	68.0	42.0
Median	23.0	16.0	19.0	15.0	71.0	20.0	92.0	59.0
75th percentile	26.0	21.0	22.0	21.0	88.0	24.0	110.3	68.0
95th percentile	28.0	27.0	24.0	27.0	101.0	27.0	125.0	78.0
Maximum observed score	28.0	28.0	24.0	28.0	105.8	28.0	133.8	83.0
Cronbach's alpha (95% confidence intervals)	0.91 (0.88, 0.94)	0.84 (0.79, 0.89)	0.87 (0.83, 0.91)	0.88 (0.84, 0.91)	0.93 (0.91, 0.95)	0.80 (0.73, 0.86)	0.94 (0.92, 0.96)	0.93 (0.91, 0.95)

Abbreviations: EWB, Emotional Well‐Being; FACT‐G, Functional Assessment of Cancer Therapy‐General; FACT‐L, Functional Assessment of Cancer Therapy‐Lung; FWB, Functional Well‐Being; LCS, Lung Cancer Subscale; PWB, Physical Well‐Being; SWB, Social Well‐Being; TOI, Trial Outcome Index.

**TABLE 4:** PROMIS‐29+2 scores.

PROMIS domain	Total sample (*N* = 2001) Mean (SD)	No Comorb. (*N* = 364) Mean (SD)	No COVID‐19 (*N* = 1920) Mean (SD)	COVID‐19 (*N* = 81) Mean (SD)
Physical function	49.7 (8.2)	55.0 (4.8)	49.8 (8.2)	47.3 (8.8)
Anxiety	53.5 (9.9)	48.7 (8.7)	53.4 (9.9)	55.7 (9.8)
Depression	52.1 (10.0)	47.2 (8.1)	52.0 (10.1)	54.8 (8.9)
Fatigue	49.4 (10.6)	42.2 (8.6)	49.2 (10.7)	52.3 (9.7)
Sleep disturbance	51.4 (9.7)	46.7 (8.9)	51.3 (9.8)	53.8 (7.3)
Ability to participate in social roles and activities	52.9 (9.7)	58.9 (7.7)	53.0 (9.6)	50.0 (10.1)
Pain interference	51.0 (9.5)	45.0 (6.3)	50.8 (9.4)	53.9 (9.9)
Cognitive function—abilities	50.4 (9.5)	51.1 (9.7)	50.6 (8.5)	47.0 (8.2)
PROPr	0.5 (0.3)	0.6 (0.2)	0.5 (0.3)	0.4 (0.2)

*Note*: PROMIS domain scores are on a *T* score metric with a mean of 50 and standard deviation of 10, referenced to the United States general population.

Abbreviations: Comorb., comorbidity; PROPr, patient‐reported outcomes measurement information system preference score; SD, standard deviation.

**TABLE 5:** Comparison of FACT‐G reference values from Brucker et al. [19] to current study.

	Brucker et al. [19],^a^ Mean (SD)	Current study, Mean (SD)
Physical Well‐Being (PWB)	22.7 (5.4)	23.1 (5.1)
Social Well‐Being (SWB)	19.1 (6.8)	16.8 (7.1)
Emotional Well‐Being (EWB)	19.9 (4.8)	18.5 (5.1)
Functional Well‐Being (FWB)	18.5 (6.8)	17.6 (6.7)
FACT‐G	80.1 (18.1)	76.0 (19.8)

^a^ Data taken from sample of 1075 adults from the US general population published in Brucker et al. [19].

Internal consistency reliability tended to exceed the threshold for good reliability (≥0.80) and, in the case of the FACT‐G, TOI, and FACT‐L Total, exceeded the threshold for excellent reliability (≥0.90). Only the LCS scale fell below the threshold for good reliability, but always exceed the threshold for acceptable reliability (≥0.70). Known‐groups validity was evidenced for the FACT‐G, LCS, TOI, and FACT‐L Total scales using each pre‐specified anchor (Table 6). For the ECOG PSR anchor, the magnitude of these differences ranged between 0.68 (medium effect) and 1.09 (large effect). Effects were smaller for the number of comorbidities anchor and were most often of medium magnitude for the PGIS anchors. One exception to that trend was for the magnitude of effects between adjacent shortness of breath PGIS groups for the LCS scale, which tended to be large.

**TABLE 6:** Group differences in FACT‐G, FACT‐L Lung Cancer Subscale (LCS), Trial Outcome Index (TOI), and Total scores.

	*N*	FACT‐G (0–108)^a^				LCS (0–28)^a^				TOI (0–84)^a^				FACT‐L Total (0–136)^a^			
		*M*	Δ	*p*	ES	*M*	Δ	*p*	ES	*M*	Δ	*p*	ES	*M*	Δ	*p*	ES
ECOG PSR																	
0	1305	82.1	‐	‐	‐	24.3	‐	‐	‐	68.9	‐	‐	‐	106.4	‐	‐	‐
1	548	68.5	13.6	<0.001	0.69	21.3	3.0	<0.001	0.68	57.1	11.8	<0.001	0.84	89.8	16.6	<0.001	0.72
2, 3, 4	148	50.3	18.2	<0.001	0.92	17.7	3.6	<0.001	0.82	41.7	15.4	<0.001	1.09	68.0	21.8	<0.001	0.95
No. of comorbidities																	
0	379	84.9	‐	‐	‐	25.0	‐	‐	‐	71.7	‐	‐	‐	109.9	‐	‐	‐
1	374	82.8	2.1	0.12	0.11	24.1	0.9	0.003	0.20	68.8	2.9	0.002	0.21	106.9	3.0	0.05	0.13
2	353	77.6	5.2	<0.001	0.26	23.4	0.7	0.04	0.16	65.2	3.6	<0.001	0.26	101.1	5.8	<0.001	0.25
3	314	75.2	2.4	0.08	0.12	22.7	0.7	0.03	0.16	62.9	2.3	0.02	0.16	97.8	3.3	0.05	0.14
≥4	581	65.3	9.9	<0.001	0.50	20.8	1.9	<0.001	0.43	54.6	8.3	<0.001	0.59	86.1	11.7	<0.001	0.51
PGIS shortness of breath																	
None	1380	80.9	‐	‐	‐	24.7	‐	‐	‐	68.3	‐	‐	‐	105.6	‐	‐	‐
Mild	420	68.1	12.8	<0.001	0.65	20.4	4.3	<0.001	0.98	56.6	11.7	<0.001	0.83	88.5	17.1	<0.001	0.74
Moderate	164	61.0	7.1	<0.001	0.36	17.2	3.2	<0.001	0.73	48.5	8.1	<0.001	0.57	78.1	10.4	<0.001	0.45
Severe/very severe	37	51.1	9.9	<0.001	0.50	13.1	4.1	<0.001	0.93	37.8	10.7	<0.001	0.76	64.2	13.9	<0.001	0.60
PGIS fatigue																	
None	562	87.9	‐	‐	‐	25.5	‐	‐	‐	73.4	‐	‐	‐	113.4	‐	‐	‐
Mild	833	79.1	8.8	<0.001	0.44	23.3	2.2	<0.001	0.50	65.8	7.6	<0.001	0.54	102.4	11.0	<0.001	0.48
Moderate	466	64.3	14.8	<0.001	0.75	20.8	2.5	<0.001	0.57	54.6	11.2	<0.001	0.79	85.1	17.3	<0.001	0.75
Severe/very severe	140	48.9	19.8	<0.001	0.78	18.0	2.8	<0.001	0.64	42.3	12.3	<0.001	0.87	66.9	18.2	<0.001	0.79
PGIS pain																	
None	788	84.8	‐	‐	‐	24.7	‐	‐	‐	71.2	‐	‐	‐	109.4	‐	‐	‐
Mild	676	76.7	8.1	<0.001	0.41	22.9	1.8	<0.001	0.41	64.0	7.2	<0.001	0.51	99.6	9.8	<0.001	0.43
Moderate	395	67.0	9.7	<0.001	0.49	21.1	1.8	<0.001	0.41	55.9	8.1	<0.001	0.57	88.2	11.4	<0.001	0.50
Severe/very severe	142	49.5	17.5	<0.001	0.88	18.8	2.3	<0.001	0.52	42.2	13.7	<0.001	0.97	68.2	20.0	<0.001	0.87

*Note*: ESs calculated as Δ/pooled standard deviation of the scale (FACT‐G = 19.8; LCS = 4.4, TOI = 14.1, Total = 23.0).

Abbreviations: Δ, adjacent category difference; ECOG PSR, Eastern Cooperative Oncology Group performance status rating; ES, effect size; *M*, mean; *p*, *p*‐value; PGIS, patient global impression of severity.

^a^ Scale theoretical (possible) range.

## 4 DISCUSSION

This study provides a comprehensive, contemporary, US general adult population‐level reference point for the FACT‐L questionnaire. It also provides further evidence for the reliability and validity of the FACT‐L. These data will have broad applicability for clinicians and researchers across the care continuum in lung cancer. As we bring novel agents into the curative setting or treat patients for longer periods of time with palliative agents, we can better assess the extent of burden related to disease or treatment over time and relative to the population at large by using measures such as FACT‐L. These data will continue to enhance our ability to inform patients around treatment decisions.

The FACT‐L reference values reported here will be useful for contextualizing FACT‐L scores from multiple types of research (e.g., clinical trials and health services) and clinical assessment. The scale means and SD in Table 3 are comparators representing the general US adult population; however, there are subsets with fewer or greater comorbidities that may apply to broader clinical populations. As an illustrative example, FACT‐L was utilized in the Phase II trial of hyperfractionated accelerated radiotherapy in Non‐small‐cell lung cancer (ECOG 4593).^20^ The FACT‐L questionnaire was administered at study entry (baseline), on the last day of radiotherapy (assessment 2), and 4 weeks after therapy (assessment 3). At baseline and the two follow‐up assessments, mean TOI scores were 60.00, 52.42, and 58.84, respectively. In comparison with the reference TOI means from the current study of 63.7 (total sample; SD = 14.1) and 72.0 (no comorbidities; SD = 10.1), it is clear that participants in ECOG 4593 were impaired, especially at the follow‐up timepoints. Particular attention might be paid to the assessment 2 score, which is more than two SD lower than the average score for respondents with no comorbidities. Information such as this may help us better understand degree of impact of therapy on HRQoL but also help us support patients during high‐risk timeframes, as highlighted in this case, which occurred after completion of therapy.

To our knowledge, this is the first study to assess FACT‐L in people who reported a diagnosis of COVID‐19. The proportion of study participants reporting a COVID‐19 diagnosis was small (4.1%), and given the self‐reporting nature of this finding, may under‐represent the true incidence of COVID‐19 in the study population. Nonetheless, participants who reported a COVID‐19 diagnosis had lower scores for all HRQoL scales compared to participants who did not report a COVID‐19 diagnosis. While it is hard to draw definitive conclusions from these findings, they may highlight novel applications for HRQOL scales in COVID‐19, such as assessing the impact of COVID‐19 infections on lung cancer patients, evaluating the duration that such an illness may affect a patient (i.e., long COVID), or evaluating degree of recovery from such an infection. There are ongoing efforts to develop a COVID‐19 specific PROM to assess HRQoL factors in patients with COVID‐19.^21^

It is important to note the strengths and limitations of having collected these data during the COVID‐19 pandemic. During the COVID pandemic, higher rates of mental health conditions were noted in the general US population in 2020 than were noted in 2019, and this may have affected our study results.^22^ The scores for PWB, SWB, and EWB in the current study were lower than those noted in prior evaluations of these scales in ambulatory oncology patients and in the general population.^16^, ^19^, ^23^ This may be reflective of the study timeframe that occurred early in the pandemic, where social distancing and “stay at home” orders were largely in place throughout the United States. These values may have less applicability for the adult populations as the pandemic improves in the future; however, the long‐term sequelae of COVID‐19 on mental health and social well‐being are still being understood and a new “normal” is still being defined.

Though this study was designed to match the joint distribution of sex and age groups within the US general adult population, it is not completely representative, and may have been in part due to the population being derived from an Internet panel. When other demographic characteristics of our sample were compared to the US general population, we found good alignment in terms of race and employment status. Yet, participants in our survey were less likely to be Hispanic or Latino, likely owing to our survey being given only in English. In addition, a lower proportion of our survey participants were married, though this is likely biased by the inclusion of an additional category of marital status, in a committed relationship, that was not featured the 2017 ACS 1‐year estimates. Finally, a great proportion of individuals participating in the survey had a university or post‐degree degree, which is expected for research participants. These differences are important to consider when interpreting our results. Nonetheless, the sample size evaluated in our study was similar to those in prior studies that established population reference values for HRQoL scales and the reference values reported in this study remain appropriate and useful for multiple research applications.^16^, ^19^, ^23^ For example, in comparison with previous studies reporting reference values from the general population for the FACT‐G subscales (PWB, SWB, EWB, FWB), the PWB and EWB scores were fairly similar to those reported here (within 1–2 points), whereas differences were larger for the SWB and FWB scales (exceeding 3 points).^16^ The integrity of the reference values from our study are further bolstered by significant evidence of reliability and validity found here, with good to excellent internal consistency reliability observed and medium to large effect sizes found for differences in FACT‐L mean scores between clinically difference participant groups.

As we are now seeing the application of PROs in different clinical settings and to monitor a variety of aspects of cancer care, this data set may have broad applicability to patients with lung cancer, at different stages of disease, and with different goals of therapy. These values allow clinicians and researchers to compare healthcare quality of life of patients with lung cancer to the general US population, allowing for better interpretation of impact of novel interventions, treatments, or patterns of care. There may be novel applications of how to use PROs, such as comparing the impact of a novel disease such as COVID‐19 to a reference population.

## AUTHOR CONTRIBUTIONS

**Nisha A. Mohindra:** Formal analysis (supporting); writing – original draft (equal); writing – review and editing (equal). **John Devin Peipert:** Conceptualization (equal); data curation (lead); formal analysis (lead); funding acquisition (supporting); methodology (equal); writing – original draft (equal); writing – review and editing (equal). **Steven I. Blum:** Conceptualization (equal); methodology (equal); writing – review and editing (equal). **James W. Shaw:** Conceptualization (equal); methodology (equal); writing – review and editing (equal). **John R. Penrod:** Conceptualization (equal); methodology (equal); writing – review and editing (equal). **David Cella:** Conceptualization (equal); data curation (equal); formal analysis (equal); funding acquisition (lead); methodology (equal).

## FUNDING INFORMATION

This study was sponsored by Bristol Myers Squibb (BMS) with a grant to Northwestern University.

## CONFLICT OF INTEREST STATEMENT

JDP reports consultation fees from consultation fees from AstraZeneca and FACIT.org; reports research grants (institutional) from Bristol Myers Squibb (BMS), Clovis Oncology, Pfizer, and Veloxis Pharmaceuticals. NAM reports consultant fees from AstraZeneca and Jazz Pharmaceuticals; reports research grants (institutional) from AstraZeneca and Pfizer. DC reports consultant fees from AbbVie, Bristol Myers Squibb (BMS), Exelixis, Merck, Novartis, and Pfizer; reports licensing fees from FACIT.org; research grants (institutional) from AbbVie, Astellas, Aveo, BMS, GlaxoSmithKline, Merck, Novartis, and Pfizer; and is an officer of FACIT.org. JWS, SIB, and JRP report employment and stock ownership with Bristol Myers Squibb.

## Supporting information


Appendix S1:

## Data Availability

The data that support the findings of this study are available on request from the corresponding author. The data are not publicly available due to privacy or ethical restrictions.
