# Supplementary material for: General population reference values for the Functional Assessment of Cancer Therapy‐Lung and PROMIS‐29
Source: Cancer Med. 2023 May 6;12(11):12765–76. doi: 10.1002/cam4.5920 (PMC10278476; doi:10.1002/cam4.5920)
Supplement: Supplementary file 1 — Appendix S1: [file CAM4-12-12765-s001.docx]

**Appendix**

| **Appendix Table 1. Demographic Breakdown of Participant Sample** | | | |
| --- | --- | --- | --- |
| **Age Group** | **Male**  **n (%)** | **Female**  **n (%)** | **Total**  **n (%)** |
| 18-19 years | 40 (2%) | 40 (2%) | 80 (4%) |
| 20-24 years | 90 (4.5%) | 90 (4.5%) | 180 (9%) |
| 25-29 years | 100 (5%) | 101 (5%) | 201 (10%) |
| 30-34 years | 80 (4%) | 80 (4%) | 160 (8%) |
| 35-39 years | 80 (4%) | 80 (4%) | 160 (8%) |
| 40-44 years | 80 (4%) | 80 (4%) | 160 (8%) |
| 45-49 years | 80 (4%) | 80 (4%) | 160 (8%) |
| 50-54 years | 80 (4%) | 80 (4%) | 160 (8%) |
| 55-59 years | 80 (4%) | 80 (4%) | 160 (8%) |
| 60-64 years | 80 (4%) | 80 (4%) | 160 (8%) |
| 65-69 years | 60 (3%) | 80 (4%) | 140 (7%) |
| 70-74 years | 40 (2%) | 60 (3%) | 100 (5%) |
| 75-79 years | 40 (2%) | 40 (2%) | 80 (4%) |
| 80-84 years | 20 (1%) | 20 (1%) | 40 (2%) |
| > 85 years | 20 (1%) | 40 (2%) | 60 (3%) |

**Survey Administration Order**

The survey was administered with two different orders for the forms. Participants were randomized to receive one of the following.

**Order 1**:

1. Sociodemographic items (7 items)
2. Comorbid Condition Checklist (23 items)
3. Eastern Cooperative Oncology Group (ECOG) performance status rating (1 item)
4. PGIS Fatigue, Pain, Shortness of Breath (3 items)
5. PROMIS Physical Function 8c 7-day short form (8 items)
6. PROMIS-29+2 Health Profile v2.1 (28 items, minus items Global 07, PFA21 and PFA23)
7. FACT-L (36 items)
8. PROMIS v1.2 Global Health (10 items)
9. PROMIS Physical Function 8c short form (8 items without 7-day context)
10. PROMIS Physical Function items PFA11 and PFA53 (2 items, with 7-day context)

**Order 2**:

- 1. Sociodemographic items (7 items)
  2. Comorbid Condition Checklist (23 items)
  3. Eastern Cooperative Oncology Group (ECOG) performance status rating (1 item)
  4. PGIS Fatigue, Pain, SOB (3 items)
  5. PROMIS Physical Function 8c short form (8 items without 7-day context)
  6. PROMIS v1.2 Global Health (10 items)
  7. FACT-L (36 items)
  8. PROMIS-29+2 Health Profile v2.1 (28 items, minus items Global 07, PFA21 and PFA23)
  9. PROMIS Physical Function 8c 7-day short form (8 items)
  10. PROMIS Physical Function items PFA11 and PFA53 (2 items, with 7-day context)
